# Determination of the ultraviolet and visible spectral response of a charge-injection device array detector

**DOI:** 10.1155/S1463924681000035

**Published:** 1981

**Authors:** H. A. Lewis, M. B. Denton

**Affiliations:** Department of Chemistry University of Arizona Tucson Arizona 85 721 USA


### Determination of the ultraviolet and visible spectral response of a charge-injection device array detector

**H.A. Lewis and M.B. Denton**
*Department of Chemistry, University of Arizona, Tucson, Arizona 85721, USA.*

### Introduction

One goal in atomic multi-elemental analysis is the ability to rapidly analyse a large number of elements over a wide concentration range. Therefore an ideal spectrophotometer should detect many wavelengths simultaneously with wide spectral coverage, good sensitivity and should exhibit a large linear dynamic range. Currently "direct readers" which employ individual slits and photomultiplier tube detectors (PMT) for each wavelength are the most commonly used. This is due to the numerous desirable characteristics of PMT's such as their high sensitivity and long, linear dynamic range. However, these instruments suffer from several significant disadvantages such as a limited number of channels, difficulty in wavelength adjustment and high cost.

Recently television-type cameras have been adapted for use as multichannel detectors in spectroscopic systems [1-3]. These devices possess a large number of picture elements (pixels) to cover a wide wavelength range rapidly and yet are compact in size. Wavelength selection can be made electronically instead of through the manual adjustment of slit-photomultiplier tube assemblies. Some of the camera devices that have been applied are silicon vidicon tubes [4-8], silicon intensified target (SIT) tubes [9-12], image dissector (ID) tubes [8,13-15], photodiode arrays (16-19), and charge-coupled device (CCD) arrays [20, 21].

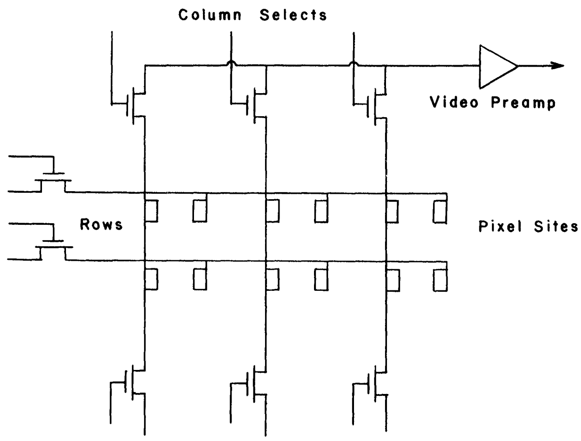

*Figure 1. Diagram of a small portion of a charge injection device array showing row and column FET selection and video preamplifier. Individual pixel (resolution) elements are comprised of a pair of metal oxide insulated capacitor plates. One on a row and one on a column. The pair of selection FETs on the columns allow separate control for voltage clamping and readout resulting in the capability to achieve the unique non-destructive mode.*

The silicon vidicon, SIT, and ID all employ an electron beam to carry the video signal. Uncertainties in the control of this beam can hamper precise pixel selection in these devices. In addition the silicon vidicon and the SIT suffer from cross-talk between pixels at high illumination levels (blooming) as well as incomplete removal of the charge signal on the target during readout (lag). In an effort to avoid these problems along with the mechanical limitations resulting from the delicate nature of vacuum tubes, solid-state cameras have been developed.

The photodiode array, CCD, and the charge-injection device (CID) [22-24] are solid state cameras that utilise sensors fabricated by employing integrated circuit fabrication technology. The photon generated charge is collected and stored in either reverse-biased photodiodes (the photodiode array) or in metal-oxide-semiconductor (MOS) capacitors (the CCD and the CID). This results in minimal lag and digitally precise pixel addressing. In general the overall package is smaller, easier to cool and less expensive than the vacuum tube cameras. To date only the photodiode array and the CCD have been applied in spectroscopic applications.

In contrast to these approaches the charge-injection device has several unique features. The CID sensor consists of a discrete two-dimensional array of pixels, each of which is composed of a pair of silicon-type MOS capacitors. Figure 1 depicts a small portion of the row/column structure with field effect transistors (FETs), etc. Light striking the bulk silicon generates charge carriers which are stored underneath the capacitor with the greatest negative potential during the frame (integration) time (Figure 2a). This can best be understood by plotting potential well curves with stored charge underneath the capacitor plates (see Figure 2b). During the readout of each pixel this charge is transferred from one of the capacitors to the other or to the substrate.

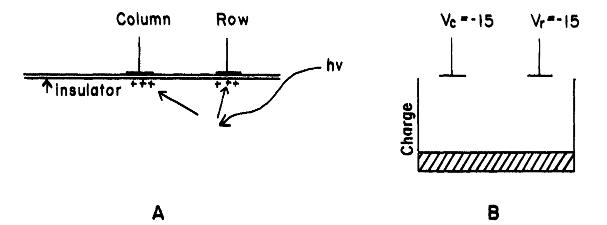

*Figure 2. (A) Photons striking the bulk silicon generate positive charge carriers which are stored beneath the negatively biased capacitor plates. (B) The quantity of charge stored beneath the plates is often depicted in a "well" diagram. When the "well" is overfilled, charge spills out and is collected by surrounding pixel sights resulting in "blooming" and other undesired "cross-talk" effects.*

Several of the important readout concepts are illustrated in Figures 3 and 4. Initially, each pixel site consisting of a row and column MOS capacitor can be thought of as having some initial bias potential V row ($V_r$) and V column ($V_C$) and an empty "well" (see Figure 3a). If both of these potentials are 15 volts negative and photons irradiate the surface, charge starts to collect in the well (see Figure 3b). If the row which crosses the particular pixel element about to be read out is clamped to zero volts, the quantities of charge stored under all the MOS capacitors within that row are transferred to each of the adjacent column electrodes (see Figure 3c). If a single column is now clamped to zero, the quantities of charge stored under all of the capacitors in the column, except for the pixel site which is in the process of being selected, merely move to their row capacitor (which is still negative).

However, for the one pixel site under selection, both the column and row capacitors are at zero potential, resulting in the stored charge being injected into the substrate (see Figure 3d). This current provides the video signal. Only the pixel at the intersection of the selected row and column has both capacitor plates at zero potential. All the other pixel sites have one of the two capacitors biased negative to store charge.

While this original mode of CID operation functions, it does not allow one of the most unique capabilities of the CID. To achieve a nondestructible readout capability, a column potential is reduced (see Figure 4a) and charge accumulated under the row capacitor. For readout, the column containing the desired pixel is allowed to float and its potential is measured. Next, the row containing the pixel is clamped to zero volts and the charge in the selected pixel moves over to its column capacitor (see Figure 4b), changing the "apparent" voltage on the entire column. This change in "apparent" column potential is dependent on the entire column capacitance and the charge accumulated in the selected pixel. If the row is returned to a more negative potential, the charge moves under it and the process can be repeated in a nondestructible readout mode, (i.e. $V_C$ is allowed to float, measured, and the processes repeated, etc.). Destructive readout is accomplished by clamping the column to zero potential while the row is still zero (Figure 4d), causing the charge to be injected, as in Figure 3d.

With a nondestructive readout capability blooming, which can still be a problem in the solid-state cameras, can be effectively eliminated by periodically scanning the array in a nondestructive readout mode to determine which pixels are near saturation. These pixels can be subsequently sampled in the destructive mode sufficiently often to prevent the leakage of charge into adjacent pixels. At the same time, pixels under low illumination can be allowed to integrate charge to maximise the signal-noise-ratio of weak lines. By averaging many repeated, nondestructive readouts of the signal stored in a pixel the readout noise, which is pre-dominately due to white noise generated in the first stage of the pre-amplifier, can be reduced by the square root of the number of complete readouts [24].

CID devices can also be fabricated to provide random addressing of the array. With this capability only the desired pixels need be interrogated instead of sequentially scanning through the whole array as is required in most other solid-state devices. Therefore, faster readout speeds are possible for rapid analysis.

On the basis of the characteristics, the CID shows definite potential as a multichannel detector for elemental analysis, provided it is able to detect light in the ultraviolet region where many elements have their strongest emission lines. The photodiode array meets this requirement but CCD's have been shown to be insensitive in this region [20] due to the absorption by the array electrodes that cover much of the sensors surface. The CID which has much smaller electrodes should have a sensitivity between that of the photodiode array and the CCD. Studies of the spectral response of CID's do not extend below 400 nm [23] or else indicate that the response quickly falls off in the UV [22]. For a number of reasons the UV response of the CID has not been measured accurately. The detector arrays are normally manufactured with a glass window. In addition the spectral response measurements were made using glass lenses [25]. Since glass absorbs light below approximately 350 nm the value of previous studies is negated. In the studies reported in this paper the spectral response for five CID sensors was measured from 190 to 800 nm using front surface mirrors and quartz optics to eliminate the absorption of glass. Five sensors were used to determine the extent of the variation in sensitivity between sensors.

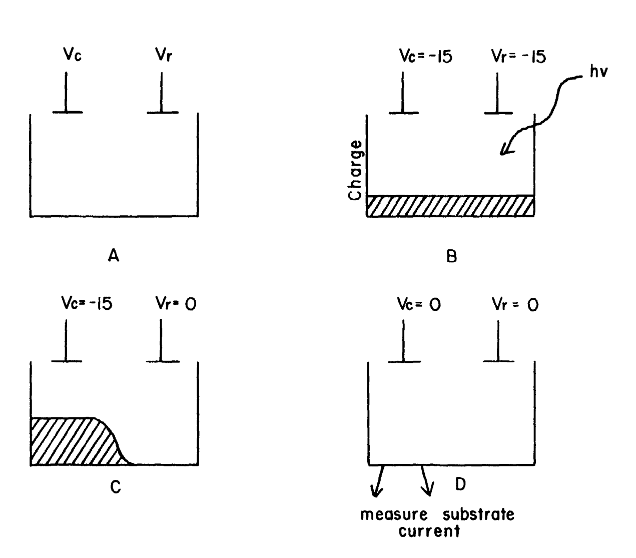

*Figure 3. The original injection mode readout for CIDs. Only the charge which is stored at the pixel site where both the column and row are clamped to zero is injected into the substrate.*

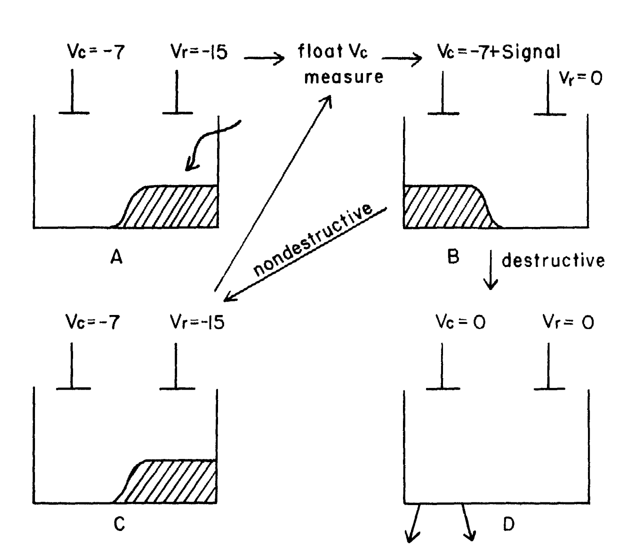

*Figure 4. The measuring sequence for combined non-destructive/destructive readout. In non-destructive mode the path B, C, float and measure is cycled through as many times as desired to improve signal to noise. (See description of operation in text)*

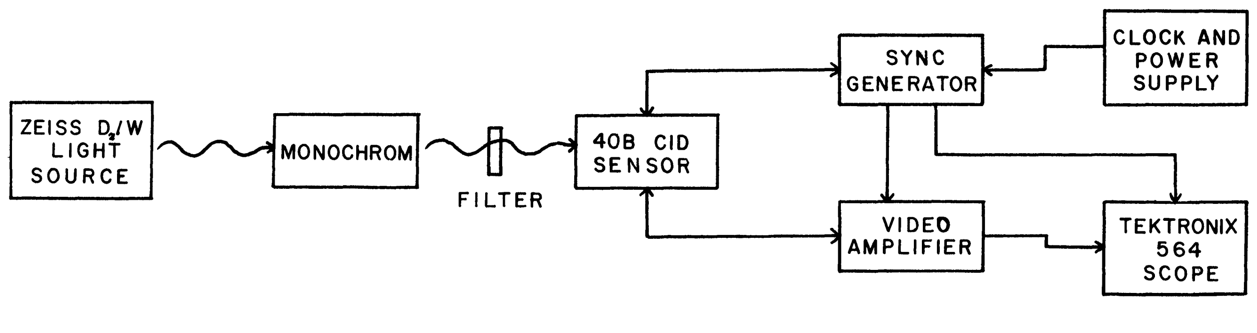

*Figure 5. Schematic diagram of the experimental system used in determining the spectral response of the CID camera.*

### Experimental

CID cameras are currently manufactured by General Electric (Syracuse, New York, USA) and are commercially available in 128 x 128, 42 x 342 and 244 x 248 pixel arrays. Five CID 40B 128 x 128 sensors were obtained from General Electric with quartz faceplates fitted instead of the standard optical glass. The faceplate covers and protects the pixel array which measures 5.85 x 5.85 mm. The sensors are mounted on 24-pin integrated circuit headers that plug into a socket on the front of a GE TN2200 camera electronics package. This electronic circuitry generates the necessary timing pulses to the chip as well as providing amplification of the video signal. It requires both a TTL clock and ± 15 volt power inputs that were supplied by a unit built in this laboratory. 40B CIDs have on-chip shift registers to provide sequential readout in a raster scan one-tenth that of the clock input. Additionally the standard TN2200 camera electronics package only provides for destructive readouts.

To determine their spectral response characteristics each sensor was evenly illuminated across the array with light from a Zeiss combination $D_2$/W lamp light source after passing through a monochromator (see Figure 5). Two different monochromators were used; a Jarrell-Ash (Waltham, Mass. USA) model #82-410 1/4 meter with 4 mm slits and a GCA/McPherson (Acton, Mass. USA) model #EU-700 0.35 meter with 2 mm slits. The Jarrel-Ash was used in the 190-400 nm region because of its greater throughput so as to provide adequate light levels for the camera in the UV. With 4 mm slits the calculated bandwidth is 13 nm. As a result of the increased sensitivity of the CID's in the visible and a stray light problem observed with the Jarrell-Ash above 500 nm the GCA/McPherson was used for the visible region measurements (400 to 800). With 2 mm slits the bandwidth of this monochromator is 4 nm.

To calibrate the irradiance level of the light striking the sensors an EG & G (Salem, Mass. USA) model #550 radiometer with a silicon photodiode probe #550-2B was used. This probe was placed in the same position as the TN2200 camera and its output read in nanoamps. The current was then converted to $\mu W/cm^2$ by use of the calibration data supplied for the probe by the manufacturer. The output voltage signal from the camera for each sensor was measured versus the wavelength setting of the monochromator with a Tektronix (Beaverton, Oregon, USA) model #564 oscilloscope. The camera generates a timing pulse after each row of 128 pixels on the array has been read out. This pulse was used to trigger the scope so that the trace displayed each row output in rapid sequence. As there was a slight variation in illumination across the rows of the array the trace waveform was observed to oscillate vertically slightly as the different rows were read. The camera reading was taken from the central pixel of the row with the greatest output to insure that the same pixel was measured each time.

The potential output was also measured for each sensor with the camera shielded from the light source (the dark potential). This potential is due to thermally generated charge in the sensor and was as much as one third of the saturation signal when low clock rates were used. For the UV measure-

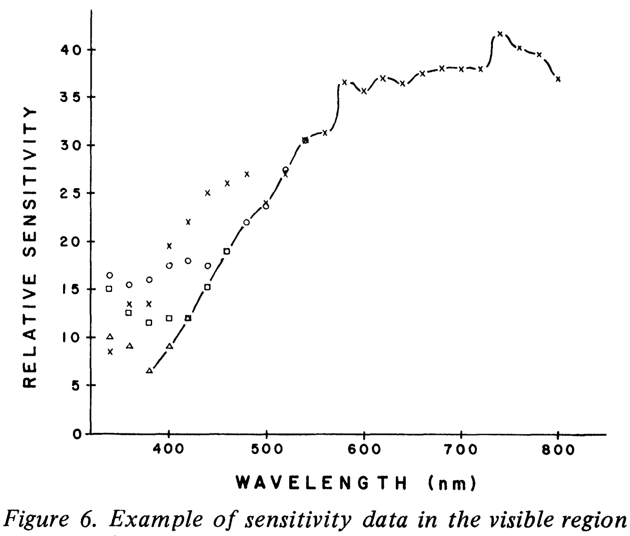

*Figure 6. Example of sensitivity data in the visible region obtained from one of the CID sensors using four different clock rates:*
*X= 1 MHz, ○= 500 KHz, □= 200 KHz, △= 100 KHz*

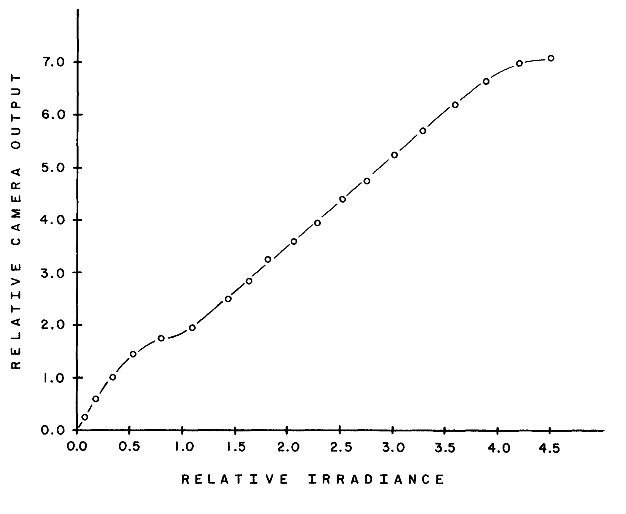

*Figure 7. Plot of the transfer curve obtained from one of the sensors.*

ments clock rates of 50 KHz, 100 KHz and 200 KHz were used depending upon the irradiance level falling on the camera. An Oriel (Stamford, Conn. USA) #772-3900 long-pass (>390 nm) filter was placed in front of the camera to determine the amount of visible wavelength stray light striking the camera. At all wavelengths except 190 nm only the dark potential was observed. The difference in signal was subtracted from measurements taken at 190 nm. Clock rates of 100 KHz, 200 KHz, 500 KHz, and 1 MHz were used for the visible region meausrements because of the greater sensitivity and hence signal at these wavelengths. All measurements were corrected for differences in integration time.

### Results and discussion

The camera sensitivity versus wavelength was calculated by dividing the net (light minus dark) camera signal by the net radiometer reading. As the proportionality factor between the amount of charge stored in a pixel and the voltage output of the TN 2200's video amplifier is unknown it was impossible to determine absolute sensitivies. Therefore only relative sensitivities are given here. The values obtained at the various clock rates were compensated for the difference in integration times by a simple multiplication factor. For example readings taken at 1 MHz were multiplied by ten to give an equivalent reading at 100 KHz. The data was then plotted as relative sensitivity versus wavelength. It was noted that in the visible region measurements taken at different clock rates would sometimes give different response values at the same wavelength even after compensation for the difference in integration times. Figure 6 is an example of this phenomena. This effect was not observed in the UV data. It was thought that non-linearity in the transfer curve (a plot of camera output versus input) of the CID's might be the cause. The transfer curves for the sensors were measured by using the GCA/McPherson monochromator set to a constant (650 nm) wavelength and varying the light intensity striking the camera by adjusting the slit widths from 0 to 2 mm. Figure 7 shows a typical curve obtained.

All of the curves showed a positive deviation from linearity at low signal levels and a slight negative deviation at high levels. The positive deviation has been observed in other CID devices [26] and can be eliminated by the use of a bias or "fat zero" charge [24]. The deviation at high signal levels has also been seen before and is thought to be due to measuring the charge at the same time it is removed (i.e. destructive readout) [23]. This should not be a problem when non-destructive readouts are used. When the non-linear regions of the transfer curve are ignored the observed variation in the sensitivity data disappear. This is illustrated in Figure 6 where a solid line connects the data that fall in the linear region. Figure 8 is a plot of the final results of the combined UV/visible data for each of the five sensors. It shows that the maximum visibile sensitivity is about seven times that of the UV region. Also, the variation in sensitivity from one sensor to the next is seen to be about ± 20% in the UV region. Assuming a typical CID sensitivity of 0.15 amps/Watt at 650 nm or a quantum efficiency of about 30% [23], the UV sensitivity would then be about 0.02 amps/Watt for a quantum efficiency in the range of 8 to 10%. It can therefore be concluded that the CID possesses a satisfactory spectral response in order to function as a multichannel spectroscopic detector.

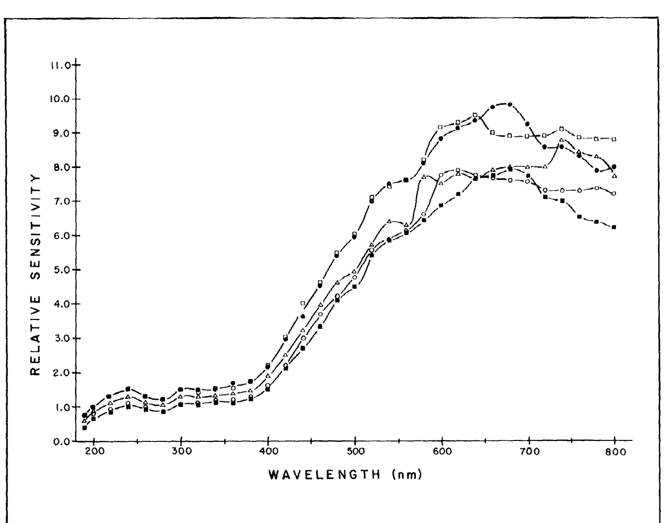

*Figure 8. Plot of combined UV/visible sensitivity data obtained from the five CID cameras. The symbols □, ■, ○, ●, and △ denote the five different sensors.*

ACKNOWLEDGEMENTS

This research was supported by a grant from the US Office of Naval Research. The authors also wish to thank the General Electric Company for the loan of several CID sensors with quartz faceplates for this study.

REFERENCES

[1] Talmi, Y., *Analytical Chemistry*, 1975, **47**, 658A.
[2] Talmi, Y., *Analytical Chemistry*, 1975, **47**, 699A.
[3] Talmi, Y., Ed., "Multichannel Image Detectors," ACS Symposium Series Vol. 102, 1979, American Chemical Society, Washington, D.C.
[4] Mitchell, D.G., Jackson, K.W., and Aldous, K.M., *Analytical Chemistry*, 1973, **45**, 1215A.
[5] Knapp, D.O., Omenetto, N., Hart, L.P., Plankey, F.W., and Winefordner, J.D., *Analytical Chimica Acta*, 1974, **69**, 455.
[6] Howell, N.G., and Morrison, G.H., *Analytical Chemistry*, 1977, **49**, 106.
[7] Felkel, Jr., H.L., and Pardue, H.L., *Analytical Chemistry*, 1977, **49**, 1112.
[8] Felkel, Jr., H.L., and Pardue, H.L., *Analytical Chemistry*, 1978, **50**, 602.
[9] Aldous, K.M., Mitchell, D.G., and Jackson, K.W., *Analytical Chemistry*, 1975, **47**, 1034.
[10] Chester, T.L., Haraguchi, H., Knapp, D.O., Messman, J.D., and Winefordner, J.D., *Applied Spectroscopy*, 1976, **30**, 410.
[11] Busch, K.W. and Malloy, B., *Analytical Chemistry*, 1979, **51**, 670.
[12] Furuta, N., McLeod, C.W., Haraguchi, H., and Fuwa, K., *Applied Spectroscopy*, 1980, **34**, 211.
[13] Golightly, D.W., Kniseley, R.N., and Fassel, V.A., *Spectrochimica Acta B*, 1970, **25**, 451.
[14] Danielsson, A. and Lindblom, P., *Applied Spectroscopy*, 1976, **30**, 151.
[15] Felkel, Jr., H.L. and Pardue, H.L. *Clinical Chemistry*, 1978, **24**, 602.
[16] Horlick, G. and Codding, E.G., *Applied Spectroscopy*, 1975, **29**, 167.
[17] Horlick, G., *Applied Spectroscopy*, 1976, **30**, 113.
[18] Yates, D.A. and Kuwana, T., *Analytical Chemistry*, 1976, **48**, 510.
[19] Chuang, F.S., Natusch, D.F.S., and O'Keefe, K.R., *Analytical Chemistry*, 1978, **50**, 525.
[20] Ratzlaff, K.L. and Paul, S.L., *Applied Spectroscopy*, 1979, **33**, 240.
[21] Ratzlaff, K.L. *Analytical Chemistry*, 1980, **52**, 916.
[22] Michon, G.J., Burke, H.K., and Brown, D.M., Proceedings of a Symposium on "Charge Coupled Device Technology for Scientific Imagining Applications", 1975, pp. 106-115.
[23] Burke, H.K. and Michon, G.J., IEEE Transactions on Electron Devices, *ED-23*, 1976, 189.
[24] Aikens, R.S., Lynds, C.R., and Nelson, R.E., Proceedings of the Society of Photo-Optical Instrumental Engineers, Vol. 78, "Low Light Level Devices", 1976, pp. 65-71.
[25] Coates P., General Electric Company, Syracuse, New York, personal communication, 1977.
[26] Aikens, R.S., Photometric, Ltd., Tucson, Arizona, personal communication, 1980.

